# Isolated Fractures of the Body of Scapula

**DOI:** 10.5704/MOJ.1311.004

**Published:** 2013-11

**Authors:** Supreeth Nekkanti

**Affiliations:** Department of Orthopaedics, Annamalai University, Tamil Nadu, India

## Abstract

Fractures of the scapula body are relatively rare constituting 1% of all fractures and 5% of all fractures around shoulder 1. They are usually caused due to high energy vehicular trauma or fall of heavy object over scapula 1,2,3. In most cases of scapula fractures, early functional treatment gives good or excellent results 1,2. Operative treatment may however be indicated in highly displaced scapular body fractures, glenoid fossa fractures, surgical and anatomical neck fractures 1,2,3,4. Very little has been published regarding the operative management of such fractures and the literature that is available, involves only a few patients 1,3. We report two patients who had isolated fracture of scapula that were treated surgically.

**Key Words:**

Isolated scapula body fracture, operative management.

## Material

Case 1: A 55-year old male was admitted to the trauma ward following a bull-gore injury. The patient was haemodynamically stable and had no other injuries. Diffuse swelling over the right shoulder and scapula was present. There was no external wound. The underlying rib cage was intact and air entry equal on both sides. The movements of right shoulder were painfully restricted. X-ray showed highly comminuted - fracture of the body of the scapula with transverse fracture of the lateral wall.

Case 2: A 27 year old male patient was admitted to emergency department due to fall of heavy object on left scapula. Patient was stable at the time of admission. Physical examination revealed tenderness over medial aspect of the left scapula. Radiographs showed surgical neck fracture extending to the medial border of left scapula.

## Method

Initially the patients were managed with an arm sling for a week. Once the swelling had subsided, surgery was planned. Under general anesthesia, the patients were placed in semiprone semiprone position. The incision extended from acromion process, curving along the spine of scapula to the inferior angle of scapula. Fracture of the neck and scapula body was visualized through this posterior approach after dissecting the infraspinatus and teres minor muscles. The suprascapular nerve and vessels entering in its superior portion were protected while reflecting these muscles. First the fracture fragments were reduced and temporarily held with Kirschner wires. The lateral border of scapula is the key structure for reduction and internal fixation of the scapular body. In the first case, the medial border was also fixed with a reconstruction plate as the fracture extended up to the medial border. In the second patient, a second plate was fixed over the infraspinatus fossa to stabilize the major fragment. Postoperatively both the patients had shoulder immobilizer and arm pouch for three weeks. Third generation cephalosporins was used for antibiotic cover. Wound healing was uneventful. After suture removal, pendulum exercises were initiated under supervision. Active range of movement exercises were started six weeks postoperative. Abduction to only 90 degrees was permitted. Gradually the range was increased and both the patients attained full range of movement by four months after surgery. The patients were followed up to one year and both had excellent outcomes in terms of pain relief, range of movements and muscle power.

## Discussion

In most cases of scapula fractures, early functional treatment gives good or excellent results 1,2. The range of motion improves over a period of time and rarely these fractures are fixed internally. The indication for internal fixation in both our patients was the extent of fracture and comminution of scapular body 1,3, 4. Small fragment 3.5 reconstruction plates were used to stabilize the fractures. More than one plate was used so that the comminuted fragments could be transfixed. In the first case, two plates were placed parallel to each other and third plate almost perpendicular to the other two plates.

In the second case, only two plates were used and they were placed parallel to each other. When the lateral border of scapula is reduced it gives us a clue for fracture reduction 3,4. However the exposure is wide and extensive. Blood loss is considerable in exposing the body of scapula. Both patients had one unit of blood transfusion per-operatively. Care is to be taken to avoid injuring the suprascapular nerves and vessels 1,4. Post-operatively the limb was kept in a sling and gentle active movements were initiated from second week. Full and pain free range was achieved by four months. At the end of a year both the patients had sound radiological union and complete functional recovery. There was no neurovascular injury in both the cases. Surgical stabilization seems to be the choice of treatment at least in severely comminuted fractures of the scapula.

**Fig. 1a:**
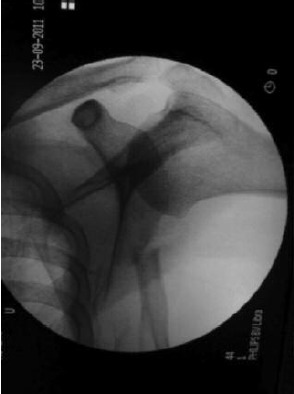
: Pre-operative radiograph of the right shoulder showing fracture of scapula body.

**Fig. 1b:**
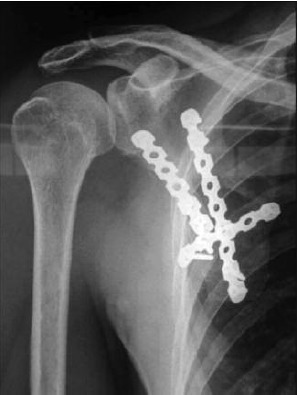
: One year post operative radiograph of the showing united fracture following plate fixation.

**Fig. 1c:**
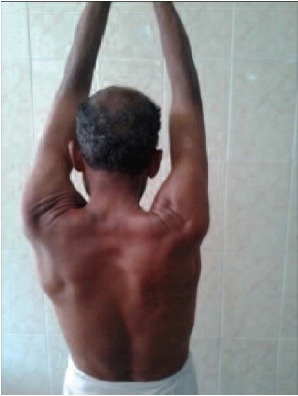
: Clinical picture of overhead abduction.

**Fig. 2a:**
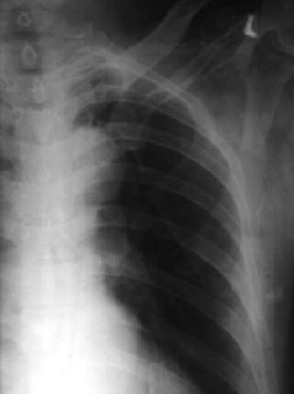
: Pre operative radiograph of the left shoulder showing scapula body fracture.

**Fig. 2b:**
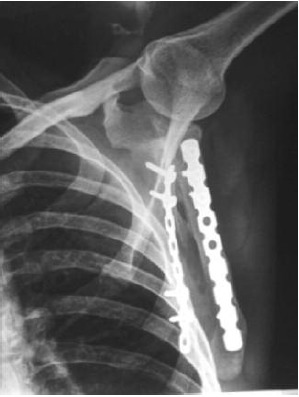
: One year post operative radiograph of the fracture following plate fixation.

**Fig. 2c:**
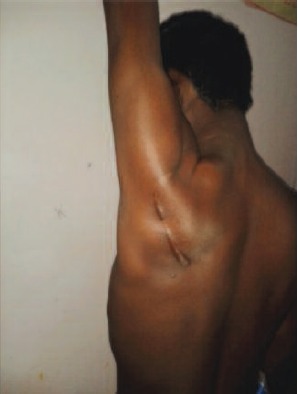
: Clinical picture of overhead abduction.

## Conclusion

Though isolated scapula body fractures are rare, operative management had given a very good outcome in both patients. Highly displaced scapula fractures can be treated surgically with predictable good functional outcomes and acceptable complication rates. The surgical management of these cases ensures early mobilization of the shoulder joint and reducing the risk of shoulder joint stiffness. The range of motion of the shoulder joint and stability is better when operated compared to what we would have expected following non operative treatment.
